# Effect of Natural Polyphenols on Breast Cancer Chemoprevention and Treatment

**DOI:** 10.1002/mnfr.70055

**Published:** 2025-04-07

**Authors:** Marzia Sichetti, Martina Giuseffi, Eugenia Giglio, Graziella Marino, Marisabel Mecca

**Affiliations:** ^1^ Laboratory of Preclinical and Translational Research Centro di Riferimento Oncologico della Basilicata (IRCCS‐CROB) Rionero in Vulture Italy; ^2^ Unit of Breast Cancer Centro di Riferimento Oncologico della Basilicata (IRCCS‐CROB) Rionero in Vulture Italy

**Keywords:** breast cancer, cancer prevention, cancer treatment, diet, polyphenols

## Abstract

Breast cancer is the most common type of malignancy among women worldwide. Significant achievements have been made in diagnostic tools and cancer treatments in the past decade; however, the complexity and heterogeneity of certain breast cancer subtypes often lead to drug resistance and metastatic progression. Owing to their low toxicity and high variety, natural products and their derivatives are becoming increasingly valuable sources for small‐molecule anticancer drugs. Polyphenols are becoming more widely known for their role in breast cancer prevention and as adjuvants in conventional treatment strategies. Therefore, this review focuses on the antitumor effects of curcumin, resveratrol, and polydatin on breast cancer. According to the main databases, only in vitro and preclinical studies with solid scientific backgrounds and reports of protective effects on breast cancer treatment were included. Curcumin, resveratrol, and polydatin have antioxidant, anti‐inflammatory, and anticancer effects; indeed, they improve drug efficacy; reduce chemoresistance, angiogenesis, and tumor growth; and induce apoptosis, autophagy, and cell cycle arrest in breast cancer through multiple molecular pathways, including the suppression of epithelial–mesenchymal transition (EMT), NF‐κB, PI3K/Akt/mTOR, c‐Jun N‐terminal kinase (JNK), MAPK, ERK1/2, and STAT signaling pathways; the inhibition of cyclins and matrix metalloproteinase (MMP)‐2 and MMP‐9; and the activation of p53 and microtubule‐associated protein light chain 3 (LC3).

## Graphical abstract

Effect of Natural Polyphenols on Breast Cancer proliferation and progression.

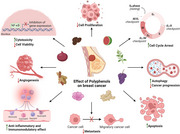

**Abbreviations**
ADP: adenosine 5’‐diphosphate
AP‐1: activator protein 1
AR: androgen receptor
ATP: adenosine 5’‐triphosphate
BAX: Bcl‐2‐associated protein x
BC: breast cancer
Bcl‐2: B‐cell lymphoma/leukemia type 2
CAT: catalase
CD: cluster of differentiation
CDC: cell division cycle
CDK: cyclin‐dependent kinase
COX: cyclooxygenase
CSCs: cancer stem cells
DHA: docosahexaenoic acid
DMSO: dimethyl sulfoxide
DNA: deoxyribonucleic acid
DOX: doxorubicin
DTX: docetaxel
ECM: extracellular matrix
EGFR: epidermal growth factor receptor
EMT: epithelial–mesenchymal transition
EPA: eicosapentaenoic acid (EPA)
ER: estrogen receptor
ERK: extracellular signal‐regulated kinase
F1,6BP: fructose‐1,6‐bisphosphate
F6P: fructose‐6‐phosphate
FA: fatty acid
G6PD: glucose‐6‐phosphate dehydrogenase
GLUT1: glucose transporter 1
HER2: human epidermal growth factor receptor 2
HIF‐1α: hypoxia‐inducible factor 1‐alpha
HO‐1: heme oxygenase 1
IAP: inhibitor of apoptosis
IC_50_
: half maximal inhibitory concentration
ICAM‐1: intercellular adhesion molecule‐1
IKKβ: inhibitory kappa B kinase beta
IL: interleukin
iNOS: induciblenitric oxide synthase 2
JAK: janus kinase
JNK: c‐Jun N‐terminal kinase
LC3: microtubule‐associated protein 1A/1B‐light chain 3
LDL: lipoproteins
LO: lipoxygenase
MAPK: mitogen‐activated protein kinase
MDR: multidrug resistance
MEK: mitogen‐activated protein kinase kinase
MMPs: matrix metalloproteinases
MPEG‐PCL: methoxypoly polycaprolactone
MPO: myeloperoxidase
mRNA: messenger RNA
mTOR: mammalian target of rapamycin
NADPH: nicotinamide‐adenine dinucleotide phosphate, reduced form
NF‐κB: nuclear factor kappa B
NLRP3: NLR family pyrin domain containing 3
NO: nitric oxide
NOx: NADPH oxidase
NQO1: NAD(P)H quinone dehydrogenase 1
Nrf2: nuclear factor 2‐related
OH: hydroxyl
PARP1: poly (ADP‐ribose) polymerase 1
PCNA: proliferating cell nuclear antigen
PFK: phosphofructokinase
PGE: prostaglandin E
pH: potential of hydrogen
PI3K/Akt: 3‐kinase/protein kinase B
POLD1: delta‐1 DNA polymerase
POLD1: DNA polymerase delta 1
PPAR‐γ: peroxisome proliferator‐activated receptor gamma
PPP: pentose phosphate pathway
PR: progesterone receptor
PUFAs: polyunsaturated fatty acids
RNA: ribonucleic acid
ROS: reactive oxygen species
SLNs: solid lipid nanoparticles
SOD: superoxide dismutase
STAT: signal transducer and activator of transcription
TGF‐β: transforming growth factor beta
TNBC: triple‐negative breast cancer
TNF‐α: tumor necrosis factor alpha
TSC2: tuberous complex of sclerosis
UV: ultraviolet
VEGF: vascular endothelial growth factor
WHO: World Health Organization
XO: xanthine oxidase

## 1 Introduction

Throughout history, the prevention and treatment of various human diseases, including breast cancer (BC), has been greatly impacted by natural products, particularly plant‐based medicines and remedies. Despite improvements in diagnostic tools and conventional treatment strategies, there are still some challenges that need to be resolved. High treatment costs, drug resistance, cellular toxicity, and frequent side effects (such as nausea, headaches, musculoskeletal pain, gastritis, oral ulcers, diarrhea, constipation, alopecia, and neuropathy) are the main causes of treatment failure and tumor relapse, leading to a poor prognosis [1, 2].

The discovery of new cancer drugs has been greatly aided by the use of polyphenols, the largest and the most diverse group of phytochemicals that are naturally found in plant‐based foods and beverages such as fruits, herbs, vegetables, teas, dark chocolate, spices, cereals, nuts and wine and many other foods in our daily diet [3].

Polyphenolic compounds have been widely acknowledged for their potential health benefits, which include antioxidant activity, immune system modulation, and anti‐inflammatory effects [4, 5, 6]. During the past few years, however, polyphenols have gained attention as potent chemosensitizing candidates with the capacity to modulate multiple cancer signaling pathways [6, 7]. There are at least 10 000 substances in the large family of polyphenols, which are constituted by one or more aromatic ring structures with single or multiple hydroxyl (OH) groups bound to them [8, 9]. Polyphenols can be classified into various groups according to the number of phenol rings, the type of linkages that join multiple rings, and the presence of carboxyl groups, natural acids, lipids, amines, or other phenolic groups. Together, these chemical elements affect the bioavailability and absorption of dietary polyphenols [4, 5, 8, 9]. Indeed, the bioavailability of polyphenolic compounds remains an important obstacle to the use of polyphenols as therapeutic agents. Usually, only a small portion of polyphenols are absorbed in the small intestine (mainly free polyphenols) and are metabolized extensively in enterocytes and the liver (through phase I and II biotransformations) [5, 10, 11]. However, the rest of the unabsorbed polyphenols reach the colon, where they are metabolized by the gut microbiota. There is a bidirectional association between the gut microbiota and polyphenols. Polyphenols affect the gut microbiome composition, promoting the growth of beneficial microbes in ways that lead to better human health. The gut microbiota metabolizes high‐molecular‐weight polyphenols into more bioactive compounds, improving their bioavailability. Because of the way polyphenols affect the microbiota, their use in medicine is now even more extensive.

BC, accounting for 2.3 million new cases a year, is the most widespread and fourth most lethal type of cancer in the world in 2022 [12, 13]. Surgery, radiotherapy, and systemic therapy, which consists of endocrine/hormone therapy, chemotherapy, targeted therapy, or a combination of these approaches, are common in BC treatment, with the goal of treating and reducing the risk of recurrence or cancer metastasis [14].

Since polyphenols have the highest number of scientific publications on cancer research and anti‐BC activity both in vitro and in vivo, our goal in this review is to present a thorough overview of the clinical evidence and recent advancements in combination/adjuvant therapy of polyphenols with conventional treatment strategies.

The combination of traditional therapies and natural products makes this new approach a cornerstone of integrated therapy. The WHO has also launched a strategic plan for 2014–2023 to promote an integrative (also called complementary) approach in the daily care of patients [15]. In 2018, the American Society of Clinical Oncology (ASCO) approved the Integrated Therapeutic Clinical Guidelines issued by the Society for Integrative Oncology (SIO) for patients with BC [16, 17]. In particular, at the hospital research institute Centro di Riferimento Oncologico della Basilicata (IRCCS‐CROB), Rionero in Vulture, Italy, the “AMICO” outpatient clinic (acronym of Ambulatorio di Medicina Integrata e Condotta in Oncologia) is taking place. Housed within the operative unit of breast surgery, the mission of the “AMICO” outpatient clinic is providing oncology‐integrated therapy recommendations for patients with BC undergoing chemotherapy and radiation therapy as well as those undergoing breast surgery.

Curcumin, resveratrol, and polydatin are currently used as adjuvant therapies or chemopreventive agents in BC therapies.

Commonly known as turmeric, *Curcuma longa* is an herbaceous plant belonging to the ginger family that was originally from India and was historically used as a natural coloring agent (food, cosmetics, and textiles), an insect repellent, and an antimicrobial agent [18]. Curcumin, the major polyphenol derived from the rhizome of curcuma, has recently attracted increased interest because of its therapeutic potential as an anti‐inflammatory and anticancer agent. Several in vitro, in vivo, and clinical trials have reported that curcumin can modulate growth factors, enzymes, transcription factors, kinases, inflammatory cytokines, and proapoptotic (by upregulation) and antiapoptotic (by downregulation) proteins [19]. There is only one major obstacle to the use of curcumin: its water insolubility and low bioavailability. These findings have led researchers to develop new formulations based on biocompatible organic substances such as liposomes, biopolymers, and cellulose to increase the therapeutic effects of curcumin.

Resveratrol is a polyphenol that can be found in peanuts, blueberries, cranberries, pistachios, and grapes. In recent years, several lines of evidence have suggested that resveratrol may be effective in the management of BC when given in combination with other chemotherapeutic or natural agents [20]. In vitro and in vivo studies have shown that this molecule suppresses BC growth, induces tumor cell death via apoptosis and autophagy, and inhibits angiogenesis, epithelial‒mesenchymal transition (EMT), and metastasis [21]. BC also acts as a multidrug resistance (MDR) reversal agent and sensitizes BC cells to chemotherapy [22].

Polydatin (also known as 3‐O‐β‐d‐resveratrol glucopyranoside) has long been used in folk medicine as a painkiller and febrifuge. The most common dietary sources of polydatin are peanuts, dairy products, chocolate (especially cocoa powder), and grapes. Absorption of polydatin can also be achieved through the diet, as in small amounts, from resveratrol by the gut microbiota [23]. Polydatin possesses a broad range of biological activities, including antioxidant, anti‐inflammatory, anticancer, and immunostimulatory effects [23]. The anticancer activity of polydatin mainly involves scavenging activity, which controls the overproduction of reactive oxygen species (ROS), leading to the suppression of some oncogenic pathways upregulated by ROS, such as the 3‐kinase/protein kinase B (PI3K/Akt) pathways, nuclear factor kappa B (NF‐κB), Wnt, matrix metalloproteinases (MMPs), and epidermal growth factor receptor (EGFR). Moreover, polydatin interferes with cyclin D1 and thus arrests the cell cycle at the G1 phase, inhibiting cancer growth.

An improved formulation is essential for the application of combined polyphenols and chemotherapy in patients. Polyphenols can be formulated into nanocarriers, such as polymer nanoparticles, micelles, nanoliposomes, polymer‒drug conjugates, dendrimers, hydrogels, nanocapsules, and exosomes, in new and promising strategies [24, 25, 26]. These formulations can ensure the effective codelivery of polyphenols and chemotherapeutics into the tumor microenvironment while simultaneously reducing toxicity and increasing drug stability.

## 2 Materials and Methods

An updated review of the role of polyphenols in the chemoprevention of BC was performed. Published studies were searched in specialized databases such as PubMed/MedLine, Scopus, Science Direct, and Google Scholar via the following keywords: “breast cancer,” “cancer prevention,” “phytochemicals,” “dietary bioactive compounds,” “polyphenols,” “resveratrol,” “curcumin,” “polydatin,” and “breast cancer cell lines” (Table 1). In particular, we focused on MCF‐7 and T‐47D cells as ER‐positive cancer models, SK‐BR‐3 cells as HER2‐positive cancer models, and MDA‐MB‐231 and MDA‐MB‐453 cells (PR‐, ER‐, and HER‐2‐negative cell lines) for triple‐negative breast cancer (TNBC) studies. Finally, MCF‐10A and MCF‐10F cells are frequently used as normal controls in BC studies.

**TABLE 1:** Summary of noncancer and malignant breast cancer cell lines and their molecular classification.

Cell lines	Organism	Immunoprofile	Characteristics
MCF‐10A	Human	ER^−^, PR^−^, HER2^−^ and EGFR^+^	Nontumorigenic epithelial cell line from mammary gland
MCF‐10F	Human	ER^−^, PR^−^, HER2^−^ and EGFR^+^	Nontumorigenic epithelial cell line from mammary gland
MCF‐7	Human	ER^+^, PR^+^, HER2^−^	Epithelial cell line from mammary adenocarcinoma
MDA‐MB‐231	Human	ER^−^, PR^−^, HER2^−^ and EGFR^+^	Epithelial cell line from mammary adenocarcinoma
MDA‐MB‐453	Human	ER^−^, PR^−^, HER2^+^ enriched and AR^+^	Epithelial cell line from metastatic mammary carcinoma
SK‐BR‐3	Human	ER^−^, PR^−^, HER2^+^ enriched	Epithelial cell line from mammary adenocarcinoma
T‐47D	Human	ER^+^, PR^+^, HER2^−^	Epithelial cell line from infiltrating ductal carcinoma

Abbreviations: AR, androgen receptor; EGFR, epidermal growth factor receptor; ER, estrogen receptor; HER2, human epidermal growth factor receptor 2; PR, progesterone receptor.

Each article was published before September 2024 and written in English, and its significance was estimated by analyzing the title and abstract. Non‐English text, lack of access to the full text, and publication in journals with no impact factor were considered exclusion criteria. Articles that met all the search criteria were evaluated in the literature and included in this review.

## 3 Polyphenols: Chemical Structure and Biosynthesis

Polyphenols are a group of organic compounds consisting of one or more benzene rings with at least one hydroxyl group (OH). The color, flavor, odor, astringency, bitterness, and oxidative stability of food can all be attributed to polyphenols [27].

Polyphenols are produced by plants as secondary metabolites, with phenylalanine and shikimic acid used as precursors. Depending on how many phenol rings they contain and the structural components that hold these rings together (such as amines, lipids, and organic and carboxylic acids), polyphenols can be categorized into several classes: tannins, phenolic acids, lignans, stilbenes, and flavonoids [27]. This high variability also results in numerous biological functions, such as antioxidant, anticarcinogenic, anti‐inflammatory, antiproliferative, and antiangiogenic properties [28, 29, 30]. Fruits (e.g., apples, berries, watermelons, and grapes), vegetables (e.g., soybeans, onions, and cereals), tea, and red wine are the main sources of polyphenols in the human diet. Some polyphenols are distinctive to certain foods, including flavanones in citrus fruit, isoflavones in soy, and phlorizin in apples, whereas others are found in all plant products, such as quercetin, which may be found in fruit, vegetables, cereals, leguminous plants, fruit juices, tea, wine, and infusions [31]. Cooking methods have a large effect on the amount of polyphenols in food. For example, since polyphenols are frequently found in greater concentrations on the outside of fruits and vegetables than on the inside, simply peeling them might remove a sizable amount of them. Moreover, it is best to steam boil veggies to prevent leaching. The content of polyphenols is strongly influenced by environmental variables [31]. These variables might involve culture in fields or greenhouses, soil type, sun exposure, and rainfall. The majority of flavonoids are significantly impacted by light exposure. Once ingested, polyphenols must be absorbed and converted into bioactive compounds, whereby they undergo enzymatic cleavage of the carbohydrate portion, and their aglycones enter small intestinal epithelial cells via passive diffusion. If they cannot be absorbed in this region, they reach the colon, where they are metabolized by the microbiota [32, 33]. The bioavailability of polyphenols varies on the basis of their class and chemical structure. According to some studies, the scale of polyphenol bioavailability varies by size from largest to smallest: phenolic acids > isoflavones > flavonols > catechins > flavanones, proanthocyanidins > anthocyanins [8, 30]. The cooking process that food goes through, pH fluctuations in the gastrointestinal tract, hydrolytic reactions by enzymes in the small intestine, phase II metabolization processes (glucuronidation, sulfation, and methylation) in the intestine and liver, and the enzymatic and catabolic activity of the intestinal microbiota all affect the bioaccessibility of polyphenols [9, 34]. A well‐known molecule belonging to the polyphenol family is resveratrol. It is often associated with several important positive biological effects on human health; unfortunately, its pharmacokinetic profile is poor. Resveratrol has decreased bioavailability because of significant metabolism by gut bacteria and phase II reactions in the gut and liver, as well as low water solubility and low chemical stability after digestion [31]. A 25 mg oral dose of resveratrol, 30 min after ingestion, results in less than 10 ng/mL of the plasma peak concentration of resveratrol [35].

## 4 Polyphenols: Biological Impact on Human Well‐Being

**FIGURE 1:**
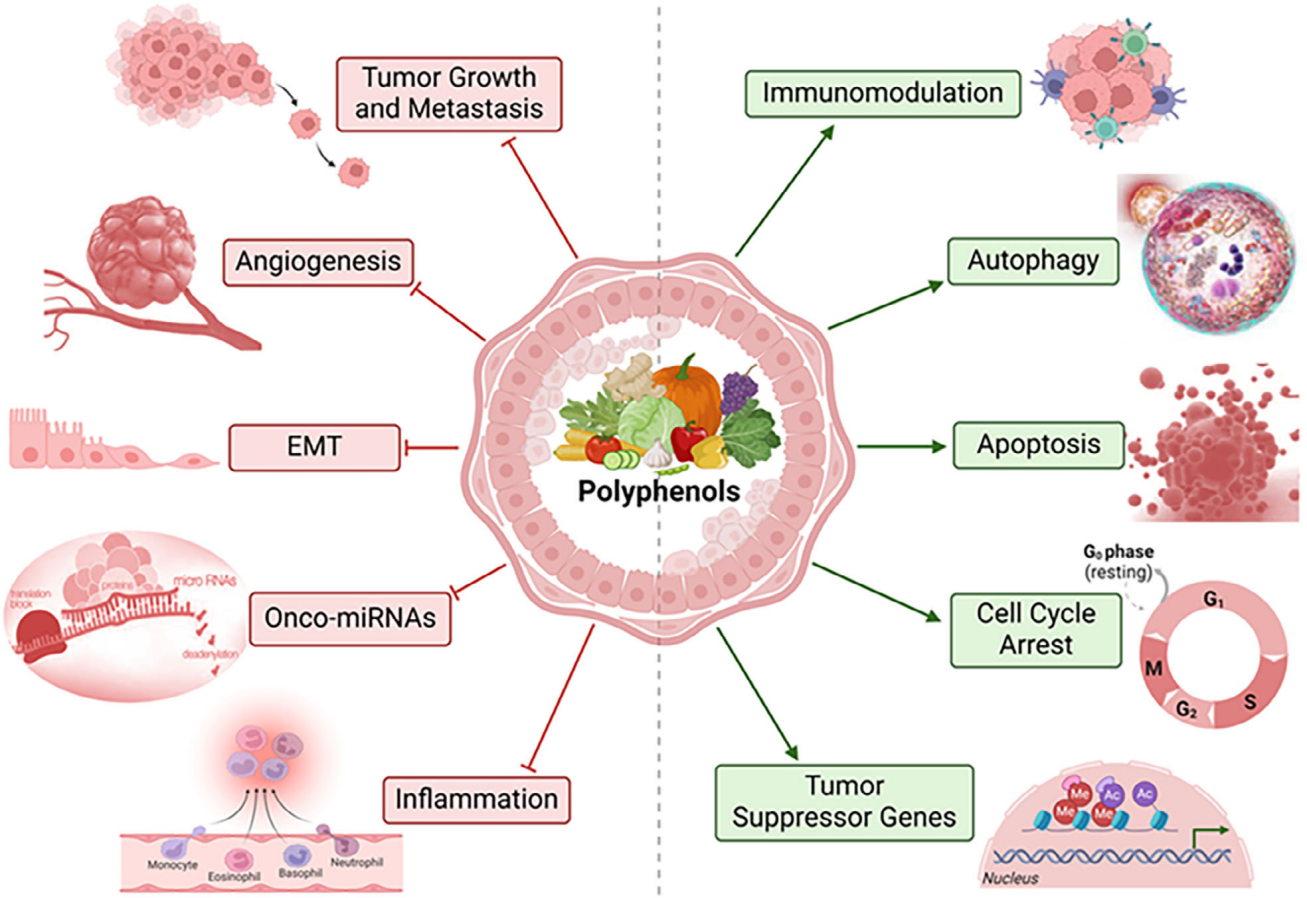
General representation of the main molecular pathways affected by polyphenols in breast cancer. The green arrows (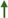) refer to promotion or increase, whereas the red hammerhead lines (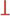) refer to inhibition. Created with BioRender.com.

Polyphenols have been shown to have anticancer properties through their ability to affect multiple cancer cell checkpoints, as shown by several studies. In particular, they are able to modulate/interfere with cell cycle arrest, proapoptotic and autophagic activation, angiogenesis, inflammation, the expression of cellular receptors or transcription factors, the inhibition of telomeres, and the synthesis of hormones [24, 36] (Figure 1).

As effective antioxidants, polyphenols can act as free radical scavengers because their phenolic groups can accept split electrons from radical molecules, transforming them into stable (less reactive) compounds [25]. In addition, they can reduce oxidative stress by inhibiting ROS‐producing enzymes such as lipoxygenase (LO), COX, myeloperoxidase (MPO), NADPH oxidase (NOx), and xanthine oxidase (XO) and by activating antioxidant enzymes such as catalase (CAT) and superoxide dismutase (SOD) [25]. For example, resveratrol increases the gene expression of the antioxidant defense enzymes NAD(P)H quinone dehydrogenase 1 (NQO1) and heme oxygenase‐1 (HO1) in cultured endothelial cells via erythroid nuclear factor 2‐related factor 2 (Nrf2), as reported by Ungvari et al. [26]. The antioxidant capacity of polyphenols makes them relevant compounds for the prevention of cancer and cardiovascular and degenerative diseases that are associated with oxidative stress.

Cell cycle dysregulation is a hallmark of tumor cells. To maintain genomic integrity after DNA damage, normal cells undergo cell cycle arrest. At the molecular level, the cell cycle is regulated by the activity of cyclin‐dependent kinase (CDK)/cyclin complexes, which govern the transition between various phases of the cell cycle. CDK alterations can contribute to tumorigenesis, so they represent a target of compounds used as anticancer drugs [37]. For example, polyphenolic compounds found in green tea and hawthorn extracts have demonstrated cytostatic activity and induce cell cycle arrest in MCF‐7 cells in the G0/G1 and S phases, respectively [38, 39].

Certain polyphenols can kill cancer cells by promoting apoptosis [3, 40, 41] and autophagy [3, 42]. Apoptosis is the main mechanism of programmed cell death and represents a key strategy for the elimination of neoplastic cells. Resveratrol induced apoptosis in MDA‐MB‐231 cells through the accumulation of nuclear COX‐2 and the phosphorylation of p53, which further triggered the expression of proapoptotic genes [3]. Moreover, resveratrol inhibited the expression of delta‐1 DNA polymerase (POLD1), which is involved in DNA replication and repair, leading to the induction of apoptosis in both in vitro and in vivo models [3]. In treated TNBC models, this was confirmed by an increase in PARP1 and caspase‐3 protein expression and a decrease in PCNA and Bcl‐2 expression [3]. Autophagy is also essential for cell survival, differentiation, development, and homeostasis [43]. Polyphenols are able to modulate the autophagy process by regulating the PI3K/Akt pathway and activating mTOR, which is the main inhibitory signal of autophagy [44].

Another important effect of polyphenols is their ability to prevent the spread of cancer cells (metastasis). Cancer cells break away from where they first form and undergo a series of physiological changes, such as loss of cell adhesion, increased expression of MMP, and endopeptidases capable of degrading extracellular matrix (ECM) proteins and promoting tumor invasion at distant sites [45, 46, 47]. Ci et al. [46] demonstrated that myricetin (belonging to the polyphenol family) has the potential to suppress BC metastasis by decreasing MMP‐2 and MMP‐9 expression and activity in a concentration‐dependent manner in MDA‐MB‐231Br cells.

Thus far, metabolism is a very important process for the survival and proliferation of cancer cells. Most of them have an altered metabolism characterized by an increase in aerobic glycolysis and lactate production to improve energy production, a phenomenon known as the Warburg effect [48]. Azevedo et al. [49] reported that some polyphenols (e.g., resveratrol) interfere with glucose absorption and the glucose transporter GLUT1 in MCF‐7 cells.

Epigenetic changes, such as DNA methylation and chromatin remodeling, regulate cell division and proliferation, making them crucial for the development of BC and the dissemination of metastatic tumors [50]. These epigenetic chromatin modifications are hereditary and reversible, so they can be used as molecular targets for new drug discovery [51]. Polyphenols have also been shown to modify DNA methylation, histone acetylation, and noncoding RNA levels, which results in epigenetic modifications in gene expression [52, 53]. Furthermore, polyphenols such as resveratrol, curcumin, and sulforaphane can modify aberrant epigenetic alterations and specifically mediate different cell signaling pathways (Wnt/β‐catenin, PI3K/Akt, and Notch) with lower levels of toxicity than other treatments for BC [50]. Zabaleta et al. [54] demonstrated that polyphenols can act on the regulation of epigenetics by modulating microRNAs associated with HER2‐positive BC.

One of the limitations in the use of polyphenols as therapeutic agents, in addition to their instability and poor solubility, is the possible interference with the absorption of nutrients or drugs [6]. For example, they can reduce the absorption of iron, thiamine, or folic acid in the body. To overcome this problem, nanotechnologies that improve absorption and bioavailability have been tested, with promising results [55]. From this perspective, various classes of polyphenols can be used as cancer treatments alone or in combination with chemotherapy and radiotherapy, resulting in greater efficacy [56].

**FIGURE 2:**
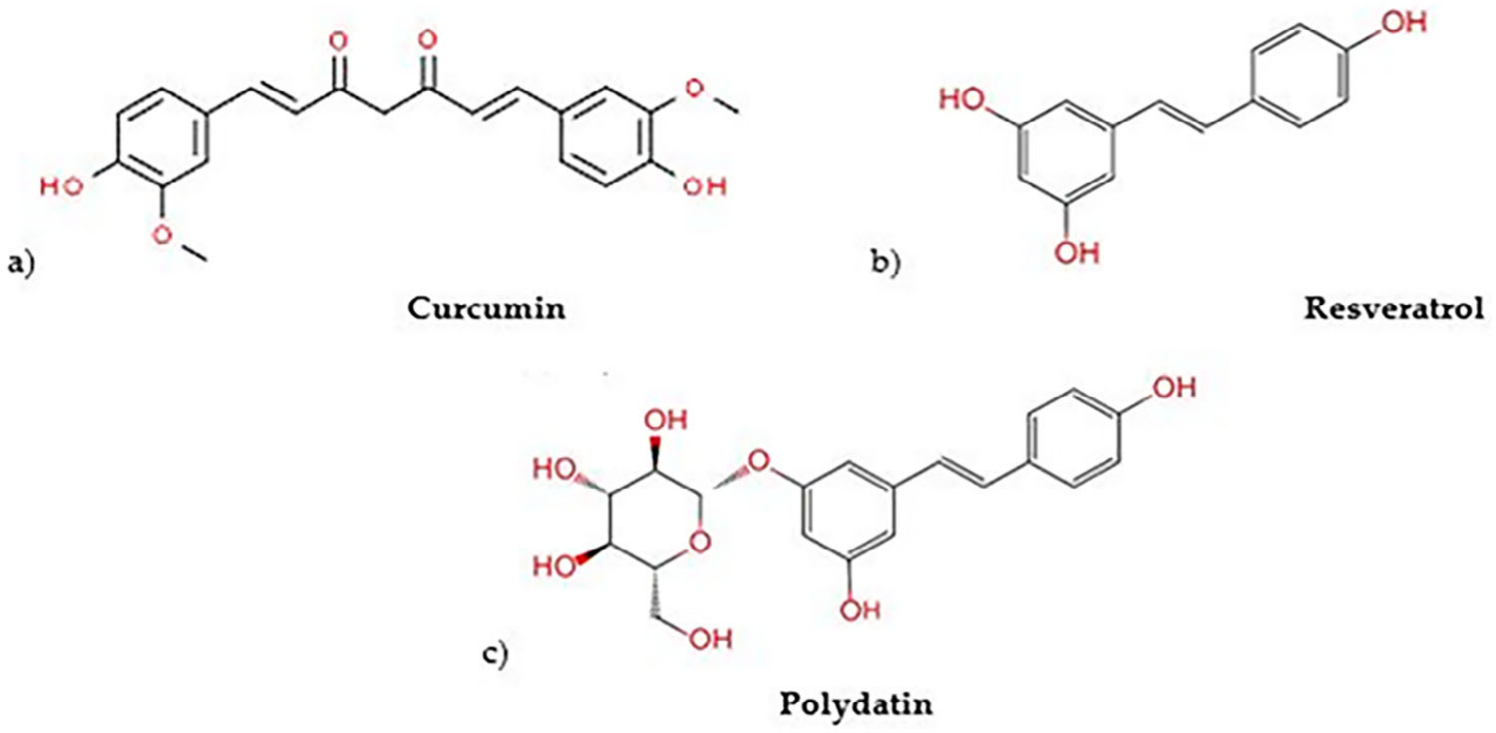
Chemical structures of (a) curcumin, (b) resveratrol, and (c) polydatin.

This review focuses on the major compounds in the polyphenol family that have exceptional antioxidant and cancer‐fighting qualities: curcumin, resveratrol, and polydatin (Figure 2).

### 4.1 Curcumin

*C. longa*, commonly known as turmeric, is an herbaceous plant belonging to the ginger family (Zingiberaceae) [57]. Turmeric is known for its bioactive polyphenolic compounds known as curcuminoids. The percentage of curcuminoids in turmeric varies between 2% and 9% depending on where it grows and the type of soil [58]. Curcumin (diferulylmethane) represents the main curcuminoid component of turmeric (approximately 77%), followed by demethoxycurcumin and bis‐demethoxycurcumin (17% and 3%–6%, respectively) and cyclocurcumin [18]. Although the extraction and separation of curcumin from turmeric powder have been known since ancient times, several polar and nonpolar organic solvents extracted followed by column chromatography have been the most commonly validated procedures [18, 58]. Curcumin and its derivatives have attracted much attention in the past two decades because of their antitumor, antioxidant, and anti‐inflammatory activities. These properties can be attributed to the chemical structure of curcumin (Figure 2a). Additionally, known as diferuloylmethane, curcumin has two phenyl rings substituted with hydroxyl and methoxyl groups connected through a seven‐carbon keto‐enol linker [58, 59, 60]. Curcumin in a mixture of keto‐enol tautomers is dependent upon the polarity and pH of the solvent [57, 59, 61]. At neutral and acidic pH values, the keto form is predominant; however, the enol tautomer is exclusively present under alkaline conditions because of the intramolecular hydrogen bonding in the enol form. Curcumin is practically insoluble at room temperature in aqueous solutions at neutral and acidic pH values [18, 57, 61]. Owing to its lipophilic nature, it is soluble in organic solvents such as methanol, ethanol, acetone, and dimethyl sulfoxide (DMSO). Different concerns should be made about the stability of curcumin in aqueous solutions, which shows a pH‐dependent trend [18, 61]. Curcumin is most stable at acidic pH values, as is the case in the stomach, where its degradation is very slow [62]. However, it is susceptible to alkaline degradation under neutral or basic conditions. Several in vitro studies have demonstrated that curcumin almost completely degrades within 30 min under physiological pH conditions and at 37°C in 0.1 M phosphate buffer and serum‐free medium [63]. Despite its low oral bioavailability and poor absorption by the gastrointestinal tract, high levels of curcumin accumulation in the small intestine have been detected [64]. These data have led many researchers to shed some light on the “low bioavailability/high bioactivity paradox” [65]. A small portion of curcumin is absorbed and rapidly metabolized in the liver by phase I and phase II enzymes and then eliminated through the gall bladder [65]. The most common part, instead, undergoes alternative metabolism by the gut microbiota [5, 64]. The human intestine and liver contain several enzymes that convert curcumin into several other metabolites, including [62, 63, 64, 65]: (i) curcumin sulfates and curcumin glucuronide, which are fairly water soluble and quickly excreted from the body via the urine and feces; and (ii) tetrahydrocurcumin, hexahydrocurcumin, octahydrocurcumin, and so forth, which have been shown to have stronger antioxidant, anti‐inflammatory, and anticancer activities than curcumin. On the basis of this evidence, the idea that diet alone is the only way to obtain an adequate amount of curcumin has been reconsidered. Some clinical studies [66, 67, 68, 69, 70] have reported that a daily intake of 12 g of curcumin is well tolerated and safe without major side effects, and the highest plasma concentrations of curcumin expressed in nanograms (ng) are usually observed in the first 1–2 h after oral ingestion. The main explanation of the challenge in figuring out the ideal dosage and the clinically meaningful effect of curcumin is its instability and degradation, together with its poor absorption, rapid metabolism, and excretion. Additionally, the majority of the studies reviewed in this review had small patient samples and were completed in a short period. By performing long‐term integration of curcumin with bigger samples, the true impact of curcumin can be determined. To counteract poor curcumin absorption and rapid elimination from the body, several drug delivery system strategies have been developed (including the use of liposomes, nanoparticles, micelles, phospholipid complexes, polymers, and adjuvants) [18, 71–73]. These novel drug delivery systems could overcome the pharmaceutical issues related to curcumin, enhancing its solubility and pharmacokinetic profile, extending its half‐life, and improving its cellular uptake. In the past few years, several studies have demonstrated the improved therapeutic potential of encapsulated curcumin in cancer treatment and its ability to inhibit cell proliferation. These data were particularly interesting when encapsulated curcumin was compared with free curcumin at the same concentration [73]. In terms of the effects of pure curcumin on human BC cell proliferation, the data revealed that treatment with different concentrations ranging from 10 to 50 µg/mL for 24, 48, and 72 h significantly decreased viability and induced apoptosis over time in a dose‐dependent manner [74]. This anti‐proliferative effect is surely related to the regulatory effects of curcumin on NF‐κB, an essential element in promoting cell survival, inflammation, differentiation, and growth. Aggarwal et al. [75] and Lui et al. [76] demonstrated that curcumin downregulated the nuclear and cytoplasmic NF‐κB pathways, which involve antiapoptotic (inhibitor of apoptosis [IAP]‐1, IAP‐2, Bcl‐2, and Bcl‐xL), proliferative (COX‐2, c‐Myc, cyclin D1, CDK4, and p21), and metastatic (vascular endothelial growth factor [VEGF], MMP‐1, MMP‐9, and ICAM‐1) proteins, respectively, in MDA‐MB‐231 and MDA‐MB‐435 cells. Curcumin interferes with the PI3K/Akt/mTOR pathway. TNBC is largely dependent on this pathway for chemoresistance and survival, which is the most frequently altered mechanism in BC [77, 78]. Curcumin inhibits signaling pathways through the downregulation of IKKβ, Akt, and HER2 expression, which may facilitate the inhibition of cellular growth, invasion, and metastasis in hormone receptor‐negative BC [60, 79]. The HER2 family has tyrosine kinase activity, and its amplification has also been correlated with STAT3 expression [80]. In two recent studies, Coker‐Gurkan et al. [81, 82] demonstrated that in MCF‐7, MDA‐MB‐453, MDA‐MB‐231, and T‐47D cells, curcumin exposure inhibited the phosphorylation of the JAK/STAT3 signaling mechanism, exerting anti‐invasive and metastatic effects with a concomitantly diminished expression of a wide range of downstream targets, such as the MAPK pathway (Ras, c‐Raf, c‐Fos, c‐Jun, c‐Myc), β‐catenin, and NF‐κB. Chung and Vadgama et al. [83] revealed that curcumin selectively blocks STAT3 phosphorylation, hence inhibiting STAT3 translocation and interaction with NF‐κB, leading to a subsequent decrease in CD44 expression, which is usually associated with the cancer stem cell (CSC) phenotype. Another major mechanism affected by curcumin is the cell cycle machinery. The cell cycle is highly regulated by several checkpoints, cyclins, and CDKs and is deregulated at multiple levels by curcumin. Zhou et al. [84] reported that the combination of curcumin and mitomycin for BC treatment enhanced G1 cell cycle arrest and inhibited tumor growth by regulating the expression levels of cyclin D1, cyclin E, cyclin A, CDK 2, CDK4, p21, and p27. Sun et al. [85] reported that low doses of curcumin induce G1 arrest in MDA‐MB‐231/HER2 cells, coupled with the downregulation of HER2, cyclin E, and CDK kinases and an increase in the p27 protein. High doses of curcumin also induce apoptosis in MDA‐MB‐231/HER2 cells and increase the expression of cleaved PARP, cleaved caspase‐3, and Bax. The opposite effect was observed for the antiapoptotic marker Bcl2. In particular, curcumin is able to induce apoptosis in a dose‐ and time‐dependent manner, accompanied by an increase in the Bax/Bcl‐2 ratio [86]. In addition, curcumin represses the metastasis of MDA‐MB‐231/HER2 cells and reduces the expression of MMP‐2 and MMP‐9 to inhibit BC cell invasion and migration. However, Hu et al. [87] reported that curcumin significantly induced cell cycle arrest at the G2/M phase, which was associated with decreases in CDC25 and CDC2 and increases in p21 protein levels in the T‐47D and MCF‐7 cell lines. An in‐depth study of this mechanism of action revealed that curcumin also induces apoptosis by inhibiting the phosphorylation of Akt/mTOR and Bcl2/Bax and the cleavage of caspase‐3, subsequently inducing the mitochondrial apoptotic pathway. In cancer cells, ROS have dual effects because they can trigger or suppress cancer cell expression. Several studies have shown that the anticancer mechanism of curcumin is mediated by the induction of ROS generation. Y. Wang et al. [88] demonstrated that curcumin is involved in ROS production. In particular, curcumin loaded in a biodegradable methoxypoly polycaprolactone (MPEG‐PCL) nanoparticle system increased the amount of cellular uptake and cytotoxicity in vitro. Furthermore, the mitochondria divided more quickly and appeared more fragmented after treatment with curcumin, which, together with a loss of mitochondrial membrane potential and a drastic increase in ROS, indicated that the degree of apoptosis of the cells increased. In addition, intravenous application of curcumin‐loaded nanoparticles inhibited the growth of MDA‐MB‐231 breast carcinoma in vivo and resulted in stronger anticancer effects than did free curcumin. W. Wang et al. [89] tested the encapsulation of curcumin into solid lipid nanoparticles (SLNs) to assess the proliferation of SK‐BR‐3 cells. After treating SK‐BR‐3 cells with free curcumin and curcumin‐SLNs, the generation of ROS was significantly increased, especially when curcumin‐SLNs were used. It has also been shown that high levels of ROS can cause depolarization of the mitochondrial membrane with a dramatically decreased Bcl‐2/Bax ratio (compared with free curcumin). Compared with free curcumin, curcumin‐SLNs have highly cytotoxic effects, probably because free curcumin enters the cell by passive diffusion, while encapsulated curcumin is constantly taken up by cells, possibly via an energy‐dependent transport pathway. Another antitumorigenic activity of curcumin is the promotion of ferroptosis, another type of programmed cell death [90]. The primary mechanism underlying ferroptosis involves the action of divalent iron or LO, which catalyzes the metabolism of unsaturated fatty acids on the cell membrane, resulting in lipid peroxidation that eventually induces cell death. Cao et al. [91] reported that curcumin dose‐dependently suppressed the viability of both MDA‐MB‐453 and MCF‐7 cells and induced ferroptosis by increasing the levels of ROS and malondialdehyde, which are the most vital end products of lipid peroxidation, and of intracellular Fe^2+^. In particular, the potential activity of curcumin as a FAS inhibitor for the chemoprevention of BC was investigated by Fan et al. [92]. As a result, curcumin induced MDA‐MB‐231 cell apoptosis by blocking FAS activity, expression, and mRNA levels in a dose‐dependent manner. Chemotherapy has become a double‐edged sword because of the significant side effects that result from the unwanted distribution of drugs, which has eventually decreased its therapeutic effectiveness. For this reason, various multifunctional or targeted drug delivery systems have been designed and prepared to enhance anticancer efficacy while reducing the side effects of chemotherapeutics on normal tissues. An important study conducted by Calaf et al. [93] highlighted how the combination of curcumin and paclitaxel had a decreased effect on apoptosis in the MDA‐MB‐231 cell line compared with that in MCF‐7 or MCF‐10F cells. In fact, curcumin and the combined treatment induced mild to greater rates of apoptosis than did paclitaxel alone in the MCF‐10F and MCF‐7 cell lines. These results indicated that either curcumin or paclitaxel alone significantly decreased NF‐κB protein expression in the MCF‐7 cell line and that combined treatment slightly decreased NF‐κB protein expression in the MDA‐MB‐231 cell line. However, paclitaxel alone increased NF‐κB protein expression in the MDA‐MB‐231 cell line, indicating possible resistance to this drug that is counteracted by curcumin when it is combined with other drugs. Calaf et al. [93] reported that curcumin suppresses NF‐κB activation, while most chemotherapeutic agents activate genes that mediate proliferation, which is why it was ascertained whether curcumin would potentiate the effect of chemotherapy in BC cell lines in the present study. The main problem with this study is that curcumin and paclitaxel are dissolved in DMSO and added to the culture medium without any prior preparation. Although it may seem old‐school, this initial trial of a therapeutic combination has laid the groundwork for more advanced and compatible formulations and delivery systems that are better suited for cells and the human body.

Liposomes have the potential to be a fascinating and promising system. Given their bilayer structure and hydrophobic properties, liposomes are closed vesicles that have the ability to spontaneously assemble. Liposomes are among the finest options for co‐delivery carriers because of their hydrophilic core and hydrophobic interlayer structure, which allow them to convey both hydrophilic and hydrophobic chemicals. Furthermore, the authors discovered that curcumin enhanced the strength of lipid membranes and reduced the passage of the encapsulated compounds [94, 95]. Ye et al. [96] evaluated the in vitro and in vivo behaviors of curcumin and docetaxel coencapsulated and delivered by liposomes. The MCF‐7 in vitro model showed that this delivery system has better sustained release effects and antitumor efficacy than free drugs do. The in vivo pharmacokinetic study indicated that the plasma concentration–time curve and biological half‐life time were significantly greater than those of free drugs, which are generally rapidly metabolized and cleared. These data confirmed that the sustained release of curcumin not only reduces side effects but also increases the anticancer activity of the drug. This liposomal delivery system was able to reverse drug resistance to docetaxel and improve the induced cytotoxicity and apoptotic effects. Kamari et al. [97] assumed that high concentrations of cisplatin modulate the proliferation of MDA‐MB‐231 cells. However, its combination with a nanoformulation of curcumin reduced the IC_50_ value of cisplatin from 58.32 to 13 µM. This lower dose of cisplatin together with curcumin still had a significant suppressive effect on the growth of MDA‐MB‐231 cells. Compared with the individual treatments, the growth inhibitory effect of the combination of curcumin and cisplatin was greater, as confirmed by the upregulation of the Bax protein and the downregulation of Bcl‐2. Several more biocompatible matrices can be utilized to create new curcumin encapsulation systems. For example, Bharmoria et al. [98] used olive oil to create an oil‐in‐water nanoemulsion. This nanoemulsion showed excellent capacity to stabilize curcumin and arrest its autoxidation. Indeed, olive oil can neutralize free radicals (such as O_2_˙ˉ, RO˙, and OH˙) and enhance the anticancer activity of curcumin in MDA‐MB‐231 cells. Kumai et al. [99] used albumin‐based lipoprotein nanoparticles to encapsulate curcumin. Albumin is a plasma protein present in the circulatory system. Various drug molecules bind to albumin, which affects its pharmacokinetics and biodistribution. It is used as a drug carrier owing to its excellent biocompatibility, high stability, high degree of drug binding, and long biological half‐life (∼19 days). It is very soluble (up to 40% w/v at physiological pH) and can resist temperatures up to 60°C. Moreover, albumin interacts with gp60 receptors, which are overexpressed in tumors; therefore, it is used as a targeted drug delivery system for cancers. The albumin‐curcumin system showed enhanced cytotoxicity due to the improved intracellular accumulation of curcumin as a result of the active targeting of albumin to cancer cells via gp‐60‐overexpressing cancer cells. Compared with free curcumin, anticancer activity was also correlated with increased ROS levels, mitochondrial membrane depolarization, and DNA damage, leading to increased cell death. Hasan et al. [100] instead worked on chitosan‐coated lecithin nanoliposomes. Lecithin nanoliposomes contain a high percentage of ω‐3 PUFAs, such as DHA and EPA, which are well known for their anticancer effects. Chitosan has been widely considered for use in bioadhesive drug delivery systems to improve the bioavailability of drugs by increasing their time at the absorption site. The membrane fluidity of these chitosan‐coated lecithin nanoliposomes strongly depends on the ω‐3 PUFA composition, whereas the presence of curcumin and chitosan decreases the membrane fluidity of all the nanoliposomes, thus increasing the rigidity of the bilayers and decreasing the movement of the FA chains. Different growth‐inhibiting effects on MCF‐7 cells were demonstrated in liposome formulations loaded with curcumin or coated with chitosan. Coating nanoliposomes with chitosan increased the loading efficiency of curcumin (88% for coated liposomes compared with 65% for noncoated liposomes) and had a stronger growth‐inhibitory effect on MCF‐7 cells. These encapsulation systems can also exploit the tumor microenvironment. Chronic inflammation is considered one of the major contributing factors to the onset and development of BC. The most studied anti‐inflammatory response of curcumin is the downregulation of COX‐2, which is involved in cytokine production and the reduction of several downstream transcription factors [101]. An example of an inflammatory mediator that has been associated with carcinogenesis is tumor necrosis factor (TNF‐α), which is involved in chronic inflammatory disease malignancy, and IL‐6, another proinflammatory cytokine that has been implicated in cancer progression by stimulating proliferation and inhibiting apoptosis [102]. In this context, Abdel‐Hakeem et al. [103] reported that, compared with free curcumin, curcumin‐loaded chitosan‐protamine nanoparticles (CU‐CHPNPs) significantly inhibited NF‐κB and proinflammatory cytokines (TNF‐α and IL‐6) in MCF‐7 cells.

In tumors, the acidity of the interstitial space and the relatively well‐maintained intracellular pH influence cancer and stromal cell function. Tumor pH is spatially and temporally heterogeneous, and cancer cells have the fitness advantage of adapting to extracellular acidity, which is particularly evident when they encounter less acidic tumor regions, for example, during invasion [104]. To address these challenges, pH‐sensitive nanoparticles have been used to improve the bioavailability of the drug in this peculiar environment, taking advantage of the pH differences between the extracellular tumor environment and normal tissue or blood. Yang et al. [105] developed a pH multistage responsive micellar system that could intelligently switch its surface charge from neutral to positive and reduce its size, thus facilitating its cellular uptake and deep tumor penetration. These micellar delivery systems were tested with a combination of paclitaxel and curcumin. The results revealed significant inhibition of proliferation in both CSCs and non‐CSCs and suppressed the formation and growth of mammospheres in vitro. Furthermore, systemic administration of the loaded micelles resulted in superior tumor inhibition activity and CSC‐killing capacity in vivo. Yuan et al. [106] exploited a similar pH‐sensitive dual drug‐loaded nanoparticle with simultaneous encapsulation of curcumin and doxorubicin (DOX). The data proved that under normal conditions (pH 7.4), the nanoparticles exhibited a cascade of sustained‐release profiles with faster release of curcumin followed by slower release of DOX. This release behavior was beneficial for enhancing the DOX distribution in tumors, thus resulting in massive killing of differentiated tumor cells.

One of the most important advantages of a co‐delivery system is the possibility to exploit the synergistic action of several drugs/molecules. The co‐delivery technology increases the substance's rate of in vivo absorption while shielding the active ingredient from the unfavorable conditions of the digestive tract. Because of the complexity and heterogeneity of the gastrointestinal system and the growing importance of the microbiota, studies conducted on cells or animals can only reveal if the delivery mechanism is safe for oral administration. But in order to replicate and predict the process of substance absorption and digestion, more accurate and sophisticated in vivo models and clinical studies need to be further investigated.

To sum up, despite its potential as an anticancer agent for BC in preclinical models, curcumin's main disadvantages (e.g., low bioavailability, solubility issues, lack of robust clinical evidence, and drug interactions) prevent it from being used as a primary treatment. Further research is needed to improve formulations and acquire stronger clinical evidence that could support the use of curcumin in the treatment of BC.

### 4.2 Resveratrol

Resveratrol (3,4′,5‐trihydroxy‐stilbene) belongs to the class of stilbenes with two phenolic rings connected by an ethylene bridge (Figure 2b) and is present in more than 70 types of plants (such as hellebores, giant polygons, grapes, and peanuts) [107]. Resveratrol can be classified into two isomeric forms, cis‐(Z) and trans‐(E), according to its chemical structure [108]. The trans isoform is the most abundant and stable isoform, even if it can be converted to a cis isomer when exposed to high pH or UV light.

The dietary items included glycosylated resveratrol. Glycosylation preserves the biological effects of resveratrol and improves its stability and bioavailability by preventing oxidation and subsequent inactivation [109]. Furthermore, glycosidases are needed for the absorption process since intestinal cells can absorb only resveratrol in its aglycone form. As a result, enterocytes can absorb only a tiny fraction of resveratrol directly, with the remainder being quickly converted by the gut microbiota into secondary metabolites that can either be absorbed or eliminated with feces [110]. When resveratrol or its metabolite finally reaches the bloodstream, due to its poor water solubility, it is complexed with low‐density lipoproteins (LDLs) or albumin and distributed throughout the body [111]. Although the biological effects of resveratrol have become widely known, its clinical application is limited by its poor water solubility (< 0.05 mg/mL), low oral bioavailability (less than 12%), short biological half‐life and rapid metabolism and elimination [112].

**FIGURE 3:**
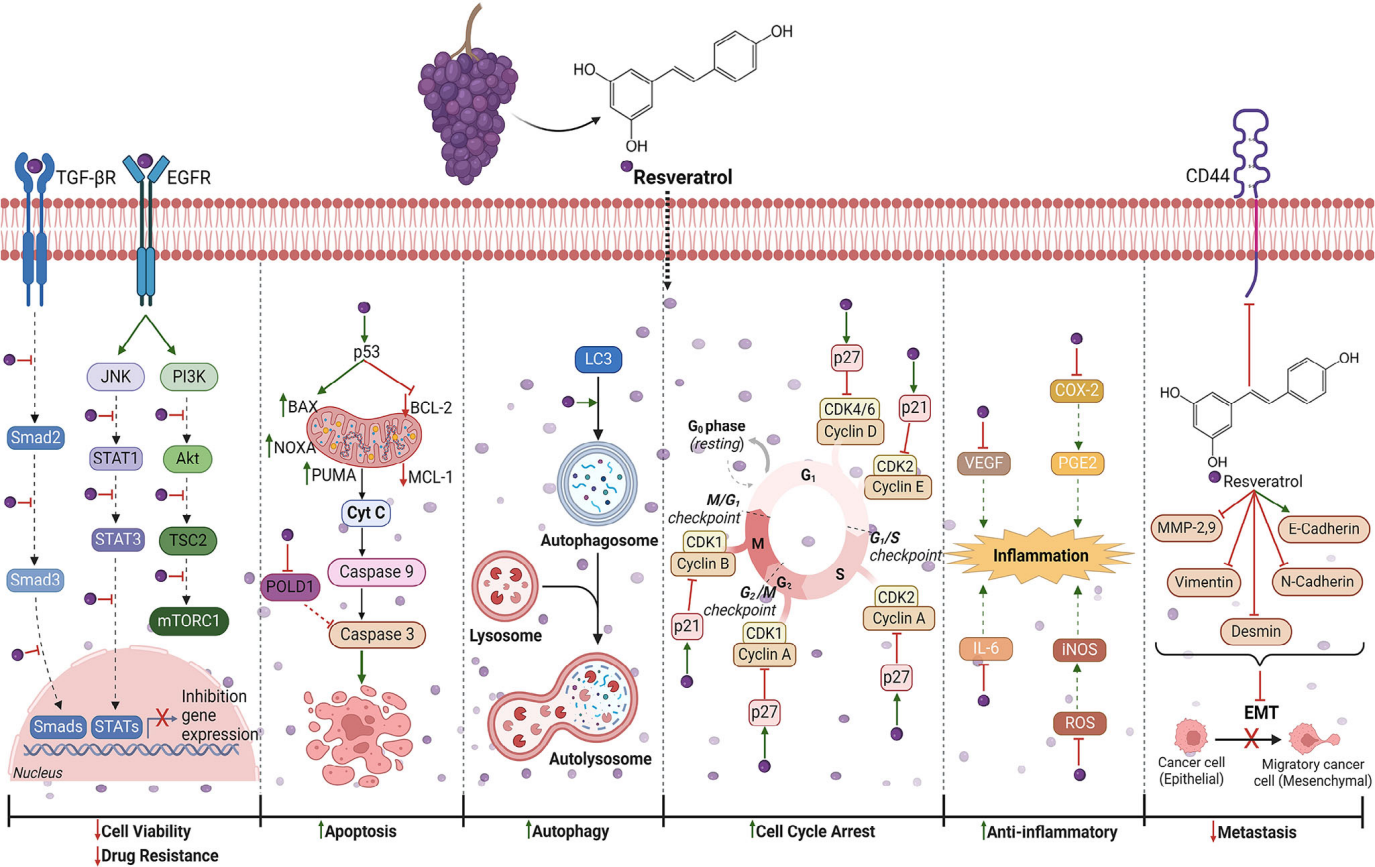
Potential molecular explanations for the antitumor effects of resveratrol. In vitro and in vivo studies have shown that resveratrol induces (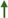) cytotoxicity and inhibits (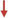) the proliferation of cancer cells primarily by (I) modulating the expression of genes crucial for cell differentiation and proliferation by transforming growth factor‐β receptor (TGF‐βR) and epidermal growth factor receptor (EGFR), inhibiting mothers against decapentaplegic homologs 2, 3 (smad2,3), c‐Jun N‐terminal kinase (JNK), signal transducer and activators of transcription 1, 3 (STAT1,3), phosphoinositide 3‐kinases (PI3K), serine/threonine protein kinase (Akt), tuberous sclerosis complex 2 (TSC2), and mechanistic target of rapamycin (mTOR); (II) inducing cell death by apoptosis via p53, Bcl‐2 associated X (BAX), NOXA, PUMA, B‐cell lymphoma 2 (BCL‐2), induced myeloid leukemia cell differentiation protein (MCL‐1), cytochrome complex (Cyt C), DNA polymerase delta 1 (POLD1), and caspases 9, 3, and by autophagy via microtubule‐associated protein light chain 3 (LC3); and (III) blocking (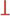) the cell cycle by inhibiting cyclins A, B, D, and E. Created with BioRender.com.

The increased interest in resveratrol was rooted in the so‐called “French paradox” in the 1990s [113, 114]. Resveratrol found in red wine is believed to play a significant role in decreasing the risk of cardiovascular disease among French citizens despite their high saturated fat intake. Since then, resveratrol has been linked to a number of pharmacological effects, especially antioxidant activity [115], as well as antiaging, anti‐inflammatory, antidiabetic, cardioprotective, and neuroprotective effects [116]. Resveratrol has gained considerable attention in the field of carcinogenesis because numerous in vitro and in vivo studies have been conducted over the years. It is thought to be useful in the fight against cancer because it can suppress the three stages of carcinogenesis (initiation, promotion, and progression) by regulating many signal transduction pathways, including those that involve cell division and growth, apoptosis, inflammation, angiogenesis, and metastasis [20, 117, 118] (Figure 3).

Tumor initiation has been linked to oxidative stress and inflammation, which can activate a variety of transcription factors, including NF‐κB, AP‐1, p53, HIF‐1α, PPAR‐γ, β‐catenin/Wnt, and Nrf2. The activation of these transcription factors can lead to the expression of more than 500 different genes, including those encoding growth factors, inflammatory or anti‐inflammatory mediators, and cell cycle regulatory molecules [21, 119]. Several studies have reported that resveratrol regulates the COX‐2/prostaglandin E3 (PGE3) inflammatory response and suppresses the production of mitochondrial ROS by downregulating the mRNA expression of inducible nitric oxide synthase 2 (iNOS) and the production of nitric oxide (NO) [120] (Figure 3). Moreover, resveratrol promotes the production of HO‐1, SOD, CAT, and NQO1 enzymes by stimulating Nrf2 phosphorylation through a PI3K/Akt‐dependent mechanism, thus causing the release of Nrf2 from Keap1 and its subsequent translocation into the nucleus [121]. Kim et al. [122] demonstrated that HS‐1793, a synthetic analog of resveratrol with improved photosensitivity and stability profiles, inhibited hypoxia‐induced HIF‐1α expression at the protein level, and its inhibitory effect was more potent than that of resveratrol in MCF‐7 and MDA‐MB‐231 BC cells. Furthermore, HS‐1793 reduces the secretion and mRNA expression of VEGF, a key mediator of HIF‐1‐driven angiogenesis, without affecting cell viability [122].

Tumor advancement involves cell proliferation and decreased death of cells. Studies conducted in vitro have revealed that resveratrol exerts anti‐proliferative effects by inducing apoptosis and modifying the balance of cyclins and CDKs, perturbing cell cycle progression. Pozo‐Guisado et al. [123] demonstrated in MDA‐MB‐231 cells that different concentrations of resveratrol (up to 200 µM) lowered the expression and kinase activities of positive G1/S and G2/M cell cycle regulators in a dose‐dependent manner without significantly affecting the expression of the tumor suppressors p21, p27, and p53. In MCF‐7 cells, resveratrol significantly and transiently increased the expression and kinase activities of positive G1/S and G2/M regulators. Moreover, p21 expression was markedly induced in the presence of high levels of p27 and p53 (Figure 3). These opposing effects resulted in cell cycle blockade at the S phase and the induction of apoptosis in MCF‐7 cells. Kim et al. [124] reported that resveratrol treatment of MCF‐7 cells resulted in dose‐dependent inhibition of cell growth and that the cells accumulated at the S phase transition. The antiproliferative effects of resveratrol were associated with marked inhibition of the cyclin D and CDK 4 proteins and induction of p53 and the CDK inhibitor p21. Growth suppression by resveratrol is also due to apoptosis, where the activation of caspase‐9, a decrease in Bcl‐2 levels, and an increase in Bax levels have been observed. Compared with those in control cells, increases in the levels of p53, p21, and Bax and parallel decreases in the expression of antiapoptotic Bcl‐2 and Bcl‐xl mRNAs were detected in MCF‐7 cells treated with resveratrol (100 µm) [125]. Liang et al. [126] studied the effects of resveratrol on the expression of genes involved in apoptosis in TNBC. In particular, the administration of resveratrol (3–200 µM) decreased the expression of DNA polymerase delta 1 (POLD1) in MDA‐MB‐231 cells in a concentration‐dependent manner but upregulated caspase‐3 and significantly decreased Bcl‐2 levels. Over the past few years, there has been a growing body of evidence indicating that resveratrol targets STAT3, PI3K/Akt/mTOR, and MAPK/ERK proapoptotic and antiproliferative signaling, which could have the potential to be used as a treatment for BC [127, 128]. Pozo‐Guisado et al. [129] reported that resveratrol was able to induce apoptosis in MCF‐7 cells via PI3K signaling and NF‐κB and Bcl‐2 downregulation. NF‐κB inhibition coincided with diminished MMP‐9 activity and cell migration. Moreover, impaired mitochondrial membrane potential and increased ROS and NOS production, which are regulators of Bcl‐2 expression, were inhibited by RES. Hu et al. [130] reported that treatment with different concentrations of resveratrol (0–400 µM) inhibited the proliferation of MCF‐7 and T‐47D cells by blocking ERK1/2, Akt, and STAT3 phosphorylation. Park et al. [131] demonstrated that resveratrol‐capped gold nanoparticles (Rev‐AuNPs) induced HO‐1 expression and markedly downregulated the nuclear translocation and transcriptional activation of NF‐κB and AP‐1 while reducing the phosphorylation of PI3K/Akt and extracellular signal‐regulated kinase ERK1/2 signaling but did not affect the phosphorylation of JNK or p38 MAPK. Furthermore, Rev‐AuNP treatment suppressed enzymatic activity and the expression of MMP‐9 and COX‐2. Nguyen et al. [132] reported that resveratrol‐induced apoptosis is correlated with sustained activation of ERK1/2 and suppression of Bcl‐2 expression. The specific inhibitor or small interfering RNA‐mediated inhibition of ERK1/2 activation reversed the effect of resveratrol on Bcl‐2 suppression and inhibited apoptosis, whereas the overexpression of MEK1, which is directly upstream of both ERK1 and ERK2, increased the degree of apoptosis induced by resveratrol. Moreover, ERK1/2 was found to act upstream of caspase‐3 to induce apoptosis, but it was not directly involved in caspase‐3 cleavage. The other closely related MAPK members, p38 and JNK, are not involved in the apoptosis induced by resveratrol in MDA‐MB‐231 cells. These results suggest that the activation of ERK1/2 is required for resveratrol‐induced apoptosis in MDA‐MB‐231 cells. Another survival mechanism used by cancer cells is the upregulation of autophagy, a conserved cellular process that maintains cellular homeostasis by degrading and recycling misfolded proteins and damaged organelles. In a very interesting experiment, Alayev et al. [133] investigated the effects of combination therapy with rapamycin (an allosteric mTORC1 inhibitor) together with resveratrol. A major challenge with the use of mTORC1 inhibitors is that rapamycin is cytostatic and not cytotoxic. mTORC1 inhibition leads to the induction of autophagy, which allows cancer cells to survive and avoid apoptosis. A consequence of mTORC1 inhibition is reactivation of Akt signaling due to suppression of the mTORC1‐mediated negative feedback loop to Akt, which over time reactivates mTORC1 signaling and is thought to contribute to drug resistance in patients. While treatment with rapamycin increased the phosphorylation of Akt, combination treatment with rapamycin and resveratrol blocked the activation of Akt. One way to measure autophagy levels is by examining LC3, whereby upon autophagy induction, LC3‐I is cleaved to LC3‐II. When MCF7 cells were treated with rapamycin, an increase in LC3‐II expression was observed, while the addition of resveratrol reduced the LC3‐II levels to near those of the control. No changes in LC3‐II were observed in immortalized nontransformed MCF‐10A cells. Similarly, MDA‐MB‐231 cells treated with rapamycin presented increased LC3‐II expression, while the addition of resveratrol reduced the degree of autophagy induction to a level lower than that of the control. Importantly, treatment of MCF7 and MDA‐MB‐231 cells with either resveratrol alone or in combination with rapamycin increased apoptosis, as shown by increased cleavage of PARP. Thus, the results indicated that the combination treatment blocks rapamycin‐induced upregulation of autophagy and induces apoptosis. Mondal et al. [134] studied the effects of resveratrol in combination with sorafenib, a multikinase inhibitor of the ERK1/2 pathway with antiangiogenic and antiproliferative activities. The data revealed an increase in intracellular ROS levels and p53 and Bax/Bcl2 expression and a decrease in the mitochondrial membrane potential. Caspase‐9, caspase‐3, and PARP were also upregulated, whereas cyclin D1 and cyclin B1 were downregulated, resulting in cell cycle arrest in the G1‒S phase. Mirzapur et al. [135], instead, tested the synergistic use of resveratrol and raloxifene, a selective estrogen receptor modulator. In MCF‐7 and MDA‐MB‐231 cells, the combination treatment impaired proliferation and viability through the signaling of Bax, p53, and Bcl2 and the expression of caspase‐3 and caspase‐8.

Tumor progression involves several processes that lead to metastasis. In this process, EMT is crucial for promoting the invasion and migration of tumor cells [136]. Tang et al. [137] demonstrated that resveratrol significantly inhibited the phosphorylation of ERK1/2 and the expression of MMP‐9 in MCF‐7 cells. One of the fundamental limitations of the literature examined so far is the inability to replicate the complexity of what happens in our body. Various systems have been developed over time, such as the Transwell system. Transwell devices have some limitations, such as the lack of a complete and physiological representation of the in vivo microenvironment, but they still offer significant advantages in many areas of biological and pharmacological research. They are essential tools for studying cell biology, metastasis, and testing new pharmaceutical compounds due to their simplicity, versatility in experimental design, and ability to analyze cell migration, invasion, and interaction mechanisms. Sun et al. [138] analyzed the effects of resveratrol on the EMT signaling pathways and the TGF‐β/Smad, PI3K/AKT, MEK/ERK, and WNT/β catenin pathways. The experiment exploited the Transwell model to evaluate the migration of TGF‐β1‐induced MDA‐MB‐231 cells. The results indicated that resveratrol reduced the secretion of MMP‐2 and MMP‐9 and increased the expression of the EMT‐related marker E‐cadherin. Moreover, decreased expression levels of MMP‐2, MMP‐9, P‐PI3K, P‐AKT, and Smad proteins and mesenchymal markers (fibronectin, vimentin, and N‐cadherin), as well as increased expression levels of E‐cadherin, were confirmed by Western blot analysis. Yang et al. [139] used the same experimental model and investigated the effect of resveratrol in combination with cisplatin. Transwell assays revealed that the combined treatment inhibited TGF‐β1‐induced cell migration and invasion, with concomitant upregulation of E‐cadherin expression and downregulation of vimentin expression. The results demonstrated that resveratrol combined with cisplatin significantly reduced the expression of P‐AKT, P‐PI3K, P‐JNK, P‐ERK, Sma2, Smad3, and NF‐κB induced by TGF‐β1. The recent focus of research has been on the ability of resveratrol to suppress glycolysis and therefore reverse the “Warburg effect.” The enzyme 6‐phosphofructose‐1‐kinase (PFK) catalyzes the irreversible conversion of fructose‐6‐phosphate (F6P) and ATP into fructose‐1,6‐bisphosphate (F1,6BP) and ADP. PFK is a highly regulated enzyme and key branching point of glycolysis, and abnormal PFK activity may be related to carcinogenesis. Gomez et al. [140] assessed the effectiveness of resveratrol in reducing ATP production and glucose metabolism in MCF‐7 cells. In addition, resveratrol reduced glucose absorption mediated by GLUT1 through direct inhibitory action. Furthermore, it is able to regulate GLUT1 expression by acting on transcription factors such as HIF‐1α and c‐Myc or by regulating various signaling pathways, such as the AMPK, Wnt, JNK kinase, and histone deacetylase pathways [141]. Thus, disrupting glucose metabolism and, consequently, the viability of BC cells. The inhibition of glycolysis could therefore represent a new goal to explain the mechanisms through which resveratrol induces anticancer effects.

MDR is the main obstacle to the effectiveness of chemotherapy treatment. Resveratrol, which interferes with different pathways involved in cell survival, helps reduce MDR [142]. The activation/deregulation of the PI3K/Akt/mTOR pathway is frequent in BC (20%–40%) and is a significant cause of aggressive tumor behavior, as well as treatment resistance, and plays a crucial role in cell viability and propagation [143]. Akt phosphorylation mediated by PI3K leads to the phosphorylation of the tuberous complex of sclerosis (TSC2), which activates mTOR, promoting the growth and metastasis of BC [144]. J. M. Chen et al. [145] confirmed that resveratrol reversed doxorubicin resistance in MCF‐7 and MDA‐MB‐231 cells, inhibiting Akt phosphorylation and upregulating PI3K. Moreover, cotreatment with doxorubicin and resveratrol inhibited cell proliferation and metastasis by inactivating the PI3K/Akt pathway and upregulating apoptosis‐dependent caspase‐3.

The therapeutic effects and promising applications of resveratrol as an anticancer agent are limited by its pharmacokinetics, which limits its therapeutic efficacy. Alkaline pH, high temperatures, UV light, and trans‐cis conversion make it a difficult compound to use due to its instability [146]. The most promising strategy is to increase the bioavailability of resveratrol by creating novel nanoformulations or resveratrol analogs, which will strengthen its anticancer effects [107, 147].

Nanotechnology is emerging as an efficient strategy to increase drug stability, therapeutic potential, and safety and reduce toxicity. Many studies have demonstrated that resveratrol can be encapsulated into various nanovectors (liposomes, lipids, polymers, and inorganic nanoparticles), leading to controlled drug release, protection against UV light, improved biostability, and sufficient therapeutic plasma concentrations [148, 149]. Gregoriou et al. [150] developed an amphiphilic triblock copolymer nanoparticle to encapsulate resveratrol, making the pharmaceutical compound fully soluble. This particular type of nanoparticle sensitizes cancer cells, increasing their vulnerability to the effects of anticancer drugs. In addition, these compounds are capable of targeting and reducing the viability of the MCF‐7 and MDA‐MB‐231 cell lines, whereas no effects have been reported in MCF‐10A cells. Wang et al. [151] formulated polyethylene glycol‐succinate‐resveratrol‐solid lipid nanoparticles (TPGS‐Res‐SLNs) to improve the therapeutic efficacy of resveratrol. The release curves of resveratrol from TPGS‐Res‐SLNs were investigated under different pH conditions, revealing that 30.27% of the resveratrol was released at 48 h in PBS (pH 7.4), whereas 45.8% of the resveratrol was released in PBS at pH 5.5. These results suggested that the TPGS‐SLNs could be utilized for controlling the release of resveratrol in a pH‐sensitive manner. Therefore, TPGS‐Res‐SLNs can increase the cellular uptake of chemotherapeutic drugs, induce mitochondrial dysfunction, and augment tumor treatment efficiency by inducing apoptosis. Moreover, SK‐BR3 cells treated with TPGS‐Res‐SLNs exhibited significant inhibition of cell migration and invasion compared with those treated with free resveratrol. Bozorgi et al. [152] synthesized chitosan nanoparticles (Cs NPs) as a resveratrol carrier in MDA‐MB 231 cells. The IC_50_ values were notably lower for the resveratrol‐loaded Cs NPs (58.246 µg/mL, 23.743 µg/mL, and 5.017 µg/mL at 24, 48, and 72 h) than for the free resveratrol (198.094 µg/mL, 111.662 µg/mL, and 118.617 µg/mL at 24, 48, and 72 h). Furthermore, resveratrol‐loaded Cs NPs had the greatest cytotoxic effect and stimulated the intrinsic apoptotic pathway. The enhanced bioavailability and biocompatibility of resveratrol delivered through nanoparticles may improve its anticancer effects and represent a valid strategy for drug delivery in difficult‐to‐treat BC. Guo et al. [153] exploited a methoxyl copolymer (mPEG‐PDLA) to study the best concentration ratios between resveratrol and docetaxel (DTX) in MCF‐7 cells. The IC_50_ values of resveratrol and DTX in MCF‐7 cells were 23.0 µg/mL and 10.4 µg/mL, respectively, whereas a lower IC_50_ of 4.8 µg/mL was obtained with the combination of resveratrol and DTX. Resveratrol‐ and DTX‐loaded mPEG‐PDLA micelles exhibited prolonged release profiles and enhanced cytotoxicity against MCF‐7 cells.

Although many studies have shown safe and well‐tolerated effects of resveratrol in in vitro, ex vivo, and animal studies, several clinical studies have reported divisive and divergent results regarding its possible toxic effects [146, 147, 148, 149, 150, 151, 152, 153, 154]. The amount of resveratrol as well as how it interacts with the redox state of the environment in which it is present can largely determine whether resveratrol will have positive or negative effects [155, 156]. Determining the precise biologically effective concentration range at which this compound should be supplemented to human subjects is challenging because of a variety of factors, including differences in the characteristics of the enrolled patients, the duration of resveratrol supplementation, and the dosage range used. In particular, in vitro, the compound's bioavailability is measured in the micromolar range in cell culture media, whereas in vivo, it is measured in the nanomolar range in blood. For example, Basheer et al. [157] and Hyrsova et al. [158] reported that when given in high amounts, resveratrol impaired P450 cytochrome activity. This may also affect the metabolism process that the medications undergo, which could result in the enzymes and drug transporters becoming less active or being overexpressed [159, 160]. In conclusion, research on the optimal resveratrol dosage that can maximize health benefits without causing toxicity issues is still ongoing.

In conclusion, preclinical and in vitro studies have shown that resveratrol has promising biological activities against BC but still has many limitations to overcome. In order to have significant therapeutic effects, it may be necessary to take high doses of resveratrol, which could lead to side effects such as gastrointestinal disorders.

Although potential anti‐cancer effects have been demonstrated in vitro and in animal studies, there is still a lack of clinical trials on humans. There are no large‐scale clinical studies that demonstrate the efficacy and safety of resveratrol as a primary or auxiliary treatment for BC. This makes it difficult to determine its real benefit in a clinical setting. Therefore, further clinical trials are essential to assess its safety and potential role in BC treatment.

### 4.3 Polydatin

Polydatin (3‐O‐β‐d‐resveratrol‐glucopyranoside) is a potent stilbenoid polyphenol extracted from the roots of *Polygonum cuspidatum*. As stated previously, polydatin is a glucoside derivative of resveratrol, in which the glucoside group linked to the C‐3 position replaces the hydroxyl group (Figure 2c), increasing its stability and solubility in water [23, 161, 162]. Polydatin is the most prevalent form of resveratrol found in nature and can be found in red wine, cocoa, and *P. cuspidatum* at an average concentration that is approximately 10 times greater than that of resveratrol [161]. Pharmacologically, polydatin and resveratrol exhibit certain similarities, but polydatin has greater bioavailability and antioxidant capabilities than resveratrol does, indicating superior cancer prevention activity and therapeutic efficiency [23, 162, 163]. Notably, the presence of a glucoside group allows polydatin to enter the cell actively via glucose transporters, in contrast to resveratrol, which passively enters the cell [164]. Furthermore, in vivo, polydatin is more resistant to enzymatic oxidation than resveratrol is; these characteristics increase polydatin bioavailability, allowing for a faster rate of absorption than resveratrol does [161]. Moreover, polydatin derivatives can be found as trans‐polydatin or cis‐polydatin isoforms, although the trans isomers display greater biological activity than the cis isomers do [162, 164].

The main biological effects of polydatin involve the modulation of signaling pathways related to oxidative stress, inflammation, and apoptosis [23, 164]. In particular, its potent antioxidant activity is linked to its chemical composition, protects it from oxidative damage caused by enzymes, and makes it more effective at neutralizing hydroxyl radicals than either vitamin C or resveratrol [165]. The impact of ROS on cancer cells can be both positive and negative and is dependent on many factors, including the type of cell, stimuli, specificity, and ROS levels. On the other hand, by directly reducing glucose‐6‐phosphate dehydrogenase (G6PD) activity, polydatin can prevent tumor development and progression by causing oxidative stress, endoplasmic reticulum stress, and death in cancer cells [166, 167, 168, 169]. The pentose phosphate pathway (PPP), a crucial route in glucose metabolism that is essential for the growth and spread of cancer, includes the key enzyme G6PD. A poor prognosis is associated with G6PD overexpression, which is observed in many human malignancies. Consequently, one of the primary goals for developing novel cancer treatments has been to block this pathway, since it has the potential to restore the susceptibility of cancer cells to chemotherapy [170]. In two distinct investigations, Mele et al. [166, 167] demonstrated how polydatin caused a redox imbalance that led to ER stress, cell cycle arrest, and death by directly inhibiting G6PD, the limiting enzyme of the PP pathway. In particular, endoplasmic reticulum stress increases sharply, and ROS accumulate when polydatin inhibits G6PD. Moreover, G6PD suppression resulted in increases in the levels of LC3B and p62, which are linked to autophagosomes and intracytoplasmic vesicles. These effects are followed by cell cycle arrest in the S phase, approximately 50% apoptosis, and 60% invasion inhibition in vitro. Zhang et al. [168] examined the anticancer effect of polydatin on the MCF‐7 BC cell line. The data demonstrated that, at 100 µmol/L, the proliferation, migration, and invasion of the treated cells were significantly reduced while apoptosis was induced. Moreover, polydatin decreased the production of MMP‐2, MMP‐9, and VEGF when it was combined with 2‐deoxy‐D‐glucose (an inhibitor of glycolysis). Moreover, combination therapy prevented the glycolytic phenotype, regulated the PI3K/AKT pathway, and reduced intracellular reactive oxygen (ROS) levels. Experiments conducted by Chen et al. [171] confirmed the involvement of this molecular pathway in the MDA‐MB‐231 and MCF‐7 cell lines. The polydatin‐induced protection mechanism is most likely the cause of the increase in phospho‐AKT, phospho‐P38, and phospho‐Erk levels. Nevertheless, the antitumor effects of polydatin and subsequent cell death cannot be overcome by this cell‐protective mechanism alone. A well‐known transcription factor called Creb (cAMP response element‐binding protein) controls several genes that have a variety of purposes, such as survival, proliferation, and the cell cycle, and plays essential roles in the growth and development of the metastasis of various solid tumors. Chen et al. [171] reported that Creb protein levels remained unchanged, whereas Creb phosphorylation levels were reduced. The inhibitory effect of polydatin may be caused by its dose‐dependent inhibition of Creb phosphorylation, which in turn suppresses cyclin D1 transcription, leading to arrest of cell cycle progression at the S phase and eventual apoptosis in BC cells. NRF2, a key antioxidant transcription factor, is one of the most important targets in BC therapy, as it frequently drives the growth of tumors. Li et al. [172] reported that the combination of brusatol (a quassinoid isolated from Brucea javanica) with polydatin significantly inhibited the proliferation of MDA‐MB‐231 and SUM159 cells in vitro. The combined therapy downregulated the protein expression of Nrf2 and downstream target genes, such as HO‐1, NAD(P)H dehydrogenase, and quinone 1 (NQO1), and increased the levels of ROS, thus strengthening the antitumor effect. Additionally, polydatin reduces the drug dosage three times in in vivo trials and has the potential to dramatically inhibit tumor cell proliferation without causing harmful side effects. This makes it a more viable therapeutic option for TNBC treatment.

Pyroptosis is a form of cell death (caspase 1‐dependent) that is triggered by proinflammatory signals and associated with inflammation. Liu et al. [173] reported that polydatin induces pyroptosis and apoptosis in TNBC by increasing the activation of caspase‐3 and lowering the phosphorylation of JAK2 and STAT3 in the cell. These findings were corroborated in a mouse model in which polydatin potently decreased distant metastases and reduced fatty liver and plasma. Moreover, polydatin increased the protein expression of caspase‐1, caspase‐3, IL‐1β, NLRP3, and IL‐18, which are involved in pyroptosis. Furthermore, the downregulation of STAT3 and JAK2 phosphorylation plays a critical role in these processes [173].

To sum up, polydatin is considered a resveratrol‐like molecule but with greater stability and bioavailability. However, despite the promising preclinical results, the use of polydatin as a BC treatment has several limitations that hinder its effective clinical application. The mechanism through which polydatin exerts its anti‐cancer effects is not yet fully understood, making it hard to optimize therapy and identify patients who could benefit more. Despite polydatin being considered safe in moderate doses, there is still a lot of uncertainty about the side effects and toxicity at high doses, so it is important to conduct a long‐term safety assessment to determine if it can be used safely as a complementary treatment for BC. In conclusion, although polydatin shows promising anti‐BC effects in preclinical studies, its limitations, including low bioavailability, incomplete understanding of mechanisms of action, and lack of robust clinical evidence, prevent its use as a standard treatment. Future research should focus on optimizing the administration of polydatin, understanding its mechanisms of action, and conducting extensive clinical trials to determine long‐term efficacy and safety.

## 5 Polyphenols Bioavailability and Availability in Clinical Practice

The difference between bioavailability and availability is crucial when considering the use of polyphenols in clinical practice [174, 175, 176]. Bioavailability refers specifically to the proportion of polyphenols that enter the bloodstream after administration and are available for therapeutic action in the body. Polyphenols, while beneficial due to their antioxidant, anti‐inflammatory, and anticancer properties, often face challenges regarding low bioavailability. This is mainly due to poor absorption in the gastrointestinal tract, rapid metabolism in the liver, and fast excretion, meaning only a small fraction reaches systemic circulation in an active form.

On the other hand, availability encompasses the total amount of polyphenols that are present at specific target sites, such as tumor tissues. This includes the bioavailable portion but also how much of the compound actually reaches the desired location in an active form, capable of exerting its therapeutic effects.

In clinical applications, enhancing the bioavailability of polyphenols is a significant challenge that directly impacts their availability for therapeutic use. This is why researchers are exploring methods like improving formulation techniques (e.g., through encapsulation, nanotechnology, or chemical modifications) to increase polyphenol absorption, prolong their half‐life in circulation, and ensure they reach their targets in an active, effective form. Without addressing these bioavailability challenges, the therapeutic potential of polyphenols remains limited, despite their promising preclinical results.

Very few clinical studies have been conducted to evaluate the antitumor effects of curcumin in humans, and currently, the available data are related to only one advanced‐stage study conducted by Saghatelyan et al. [177]. In a double‐blind randomized controlled clinical trial, women with advanced metastatic BC received treatment with paclitaxel with placebo or paclitaxel with curcumin (300 mg solution) via intravenous administration for 12 weeks with 3 months of follow‐up. The paclitaxel‐curcumin combination produced a superior response compared to the placebo. Moreover, intravenous curcumin reduced fatigue and did not have adverse effects on the patients’ quality of life, suggesting better tolerance in the curcumin group.

The heterogeneity of studies and variable characteristics, such as different types of administration and dosages of curcumin, lead to poor comparability of the data [178, 179]. To overcome its limitations and ensure its use in clinical practice, appropriate dosage forms and duration of curcumin use should be determined, also considering the potential risks of higher dosages. Moreover, the heterogeneity of curcumin formulations, as well as their pharmacokinetic variability and use in individuals with locally advanced or metastatic tumors, may have also contributed to an underestimation of their potential clinical effect.

Similarly, resveratrol has shown chemopreventive properties in several studies conducted mainly on murine models, demonstrating a reduced susceptibility of mice to BC or reducing lung metastases without having any effect on body weight or key organs [180]. However, despite its promising antitumor activities, the poor bioavailability of resveratrol (about 1%) after oral ingestion significantly limits its therapeutic application, especially in organs distant from the gastrointestinal tract [181, 182]. For this reason, different studies have been focused on the role of this molecule in the prevention of colorectal cancer. Patel et al. [183] demonstrated that resveratrol, given to patients with colorectal cancer, can be easily absorbed through the digestive tract after oral intake, particularly in high doses, with a positive outcome for these patients.

It is a challenge to determine the optimal approach of administration to guarantee resveratrol's high bioavailability [184], which could lead to its clinical use as a BC chemopreventive agent. In an interesting study, the different effects of resveratrol were evaluated in powder and soluble form by Ren et al. [185]. Resveratrol was significantly more prevalent in the blood plasma of the volunteers to whom the soluble form had been administered than the powdered form. Confirming the soluble formulation of resveratrol's superior absorption and tolerability. To overcome the bioavailability/availability limitations, recent research has focused on the customization of smart carriers, such as the development of targeted nanohydrogels capable of sequestering resveratrol in normal tissue and releasing it at the tumor site, protecting it from its rapid metabolism and maximizing its accumulation in cancerous tissue [186]. Furthermore, a considerable number of natural and synthetic analogs of cis‐ and trans‐resveratrol are currently being tested with the aim of improving the bioavailability of the compound. For instance, W. Zhu et al. [187] investigated how resveratrol affected prostaglandin E2 (PGE2) expression and DNA methylation in a group of women at high risk for BC. In this double‐blind randomized study, participants were divided into three groups and given a capsule twice a day for 12 weeks that contained either a placebo, 5 mg of trans‐resveratrol, or 50 mg of trans‐resveratrol. The study found a suppression of DNA methylation of a BC‐related gene and a lowering of the cancer‐promoting PGE2 in a dose‐dependent manner. The use of polydatin in clinical trials is even rarer, with two more recent studies investigating its use in the treatment of pediatric irritable bowel syndrome [188, 189].

Currently, there are many more published articles on preclinical investigations of resveratrol and polydatin than there are on clinical research. The underlying causes of this phenomenon include a variety of characteristics that may make it more challenging to adapt preclinical findings in clinical settings, such as differences in patient populations, variations in disease progression, the complexity of human biology and genetics, and differences in drug metabolism, all of which can contribute to inconsistent results when translating from laboratory settings to real‐world clinical applications.

While many preliminary clinical studies have explored the use of polyphenols in BC treatment, most have been limited in terms of size, duration, or methodology. The variety of approaches used, coupled with the difficulty in standardizing polyphenol dosing, makes it challenging to establish clear guidelines for their use as anticancer therapy in clinical trials. Variability in the quality and concentration of polyphenol preparations, along with the diversity of formulations (e.g., capsules, powders, liquids), further complicates the determination of an optimal dose. Additionally, the challenge of attributing chemopreventive effects to polyphenols is linked to the fact that most human studies have used products or extracts containing different polyphenols, either alone or in combination. This variability makes it difficult to pinpoint which specific polyphenols are responsible for the observed antitumor effects. Therefore, it is essential to account for variations in the polyphenolic composition of plant foods, as well as their interactions with other food components, since these factors inevitably influence their bioavailability and potential chemopreventive effects.

Furthermore, the response to treatment can vary considerably between individuals, complicating the evaluation of its efficacy in a larger population. In particular, the interaction between polyphenols and the intestinal microbiota must be considered, as it contributes to the high interindividual variability in treatment responses [190, 191, 192]. Polyphenols, such as resveratrol and curcumin, have well‐documented antitumor effects in preclinical models, but their therapeutic efficacy in humans is limited by low bioavailability due to rapid metabolism and poor solubility. This is where the microbiota comes into play: certain gut bacteria can convert polyphenols into active metabolites, which may enhance their therapeutic effects. Recent studies have shown that the activity of polyphenols can vary greatly from individual to individual due to differences in microbiota composition, contributing to the variability in treatment outcomes. In the context of BC, the interaction between polyphenols and the intestinal microbiota may influence the modulation of inflammation, regulation of the immune system, and activation of molecular pathways involved in tumor growth and metastasis. Therefore, a better understanding of how the microbiota influences the absorption and efficacy of polyphenols is essential for optimizing the use of these compounds as adjunctive therapies. Additionally, personalizing treatment based on each patient's microbiota profile could improve the efficacy of polyphenols in BC therapy. In conclusion, the interaction between the intestinal microbiota and polyphenols represents a promising frontier in personalized medicine for BC. Future studies should focus on identifying microbiota markers that predict responses to polyphenols to enhance the use of these compounds in oncology treatments.

## 6 Conclusions and Future Prospects

Owing to their exceptional anti‐inflammatory, antitumor, and antioxidant qualities, medicinal plants and herbs with pharmacological importance have attracted increasing interest in recent decades. Preclinical research on a variety of cancer lines and experimental animal models has shown the efficacy of polyphenols, either individually or in combination with chemotherapeutic drugs, which work via distinct molecular pathways. Polyphenols, particularly curcumin, resveratrol, and polydatin, have demonstrated multitarget ‘pleiotropic’ effects that may disrupt these oncogenic signaling pathways, which regulate different stages of the development of BC, including the NF‐κB, PI3K/Akt/mTOR, JNK, JAK/STAT, MAPK, and ERK1/2 pathways; EMT and MMP‐2 and MMP‐9; p53 and microtubule‐associated protein light chain 3 (LC3); and cell cycle arrest, oxidation state, and angiogenesis.

Despite the positive outcomes, questions remain about the optimal dosage, potential side effects, and types of formulations. Additionally, while preclinical models suggest that polyphenols may enhance the effects of conventional therapies (such as chemotherapy and radiation), the precise mechanisms of action remain unclear. Polyphenols, as natural compounds, tend to have fewer side effects and lower toxicity compared to traditional medications, making them a valuable complement to conventional treatments. However, it is essential to keep in mind that some polyphenols may exhibit opposing effects.

Therefore, more clinical investigations are needed to confirm and improve the benefits of polyphenols for cancer patients and to provide essential data on how polyphenols can improve patient outcomes. The future use of polyphenols in clinical trial settings will require an ever‐increasing number of well‐designed clinical trials where a multidisciplinary team, including oncologists and researchers, is indispensable to advancing polyphenols as safe, effective, and scientifically validated tools in cancer treatment. Collaborative efforts will be crucial to optimize dosing strategies, monitor patient responses, and overcome challenges such as bioavailability.

In conclusion, polyphenols are predominantly endorsed for cancer treatment because of their safety, straightforward accessibility, and anti‐cancer potential; however, to link their usage with the conventional BC treatment protocol, more research is needed.

## Conflicts of Interest

The authors declare no conflicts of interest.

## Peer Review

The peer review history for this article is available at https://publons.com/publon/10.1002/mnfr.70055.
